# Human mesenchymal stromal cells inhibit tumor growth in orthotopic glioblastoma xenografts

**DOI:** 10.1186/s13287-017-0516-3

**Published:** 2017-03-09

**Authors:** Simone Pacioni, Quintino Giorgio D’Alessandris, Stefano Giannetti, Liliana Morgante, Valentina Coccè, Arianna Bonomi, Mariachiara Buccarelli, Luisa Pascucci, Giulio Alessandri, Augusto Pessina, Lucia Ricci-Vitiani, Maria Laura Falchetti, Roberto Pallini

**Affiliations:** 1 Institute of Neurosurgery, Università Cattolica del Sacro Cuore, Rome, Italy; 2 Institute of Anatomy, Università Cattolica del Sacro Cuore, Rome, Italy; 3 Department of Biomedical, Surgical and Dental Sciences, University of Milan, Milan, Italy; 4 Department of Cerebrovascular Diseases, Fondazione IRCCS Neurological Institute Carlo Besta, Milan, Italy; 5 Department of Hematology, Oncology and Molecular Medicine, Istituto Superiore di Sanità, Rome, Italy; 6 Department of Veterinary Medicine, University of Perugia, Perugia, Italy; 7 CNR-Institute of Cell Biology and Neurobiology (IBCN), Rome, Italy

**Keywords:** Human mesenchymal stromal cells, Glioblastoma, Orthotopic tumor xenograft

## Abstract

**Background:**

Mesenchymal stem/stromal cells (MSCs) represent an attractive tool for cell-based cancer therapy mainly because of their ability to migrate to tumors and to release bioactive molecules. However, the impact of MSCs on tumor growth has not been fully established. We previously demonstrated that murine MSCs show a strong tropism towards glioblastoma (GBM) brain xenografts and that these cells are able to uptake and release the chemotherapeutic drug paclitaxel (PTX), maintaining their tropism towards the tumor. Here, we address the therapy-relevant issue of using MSCs from human donors (hMSCs) for local or systemic administration in orthotopic GBM models, including xenografts of patient-derived glioma stem cells (GSCs).

**Methods:**

U87MG or GSC1 cells expressing the green fluorescent protein (GFP) were grafted onto the striatum of immunosuppressed rats. Adipose hMSCs (Ad-hMSCs), fluorescently labeled with the mCherry protein, were inoculated adjacent to or into the tumor. In rats bearing U87MG xenografts, systemic injections of Ad-hMSCs or bone marrow (BM)-hMSCs were done via the femoral vein or carotid artery. In each experiment, either PTX-loaded or unloaded hMSCs were used. To characterize the effects of hMSCs on tumor growth, we analyzed survival, tumor volume, tumor cell proliferation, and microvascular density.

**Results:**

Overall, the AD-hMSCs showed remarkable tropism towards the tumor. Intracerebral injection of Ad-hMSCs significantly improved the survival of rats with U87MG xenografts. This effect was associated with a reduction in tumor growth, tumor cell proliferation, and microvascular density. In GSC1 xenografts, intratumoral injection of Ad-hMSCs depleted the tumor cell population and induced migration of resident microglial cells. Overall, PTX loading did not significantly enhance the antitumor potential of hMSCs. Systemically injected Ad- and BM-hMSCs homed to tumor xenografts. The efficiency of hMSC homing ranged between 0.02 and 0.5% of the injected cells, depending both on the route of cell injection and on the source from which the hMSCs were derived. Importantly, systemically injected PTX-loaded hMSCs that homed to the xenograft induced cytotoxic damage to the surrounding tumor cells.

**Conclusions:**

hMSCs have a therapeutic potential in GBM brain xenografts which is also expressed against the GSC population. In this context, PTX loading of hMSCs seems to play a minor role.

**Electronic supplementary material:**

The online version of this article (doi:10.1186/s13287-017-0516-3) contains supplementary material, which is available to authorized users.

## Background

Mesenchymal stem/stromal cells (MSCs) were first defined by Friedenstein et al. [1] as bone marrow (BM)-derived, adherent, fibroblast-shaped cells with the capacity to differentiate into bone. These cells were later demonstrated to have the ability to self-renew, form colonies, and differentiate toward different mesodermal cell types [2]. Three minimal criteria have been established to define MSCs: 1) to adhere to plastic in vitro; 2) to exhibit a set combination of surface markers (CD73^+^, CD90^+^, CD105^+^, CD44^+^, CD166^+^, CD34^–^, CD31^–^, CD40^–^, CD45^–^, CD14^–^, and HLA-II^–^); and 3) to differentiate in vitro into osteoblasts, chondrocytes, and adipocytes [3]. Beside these minimal standards, further criteria for MSC identification may vary, making it difficult to strictly define MSCs. MSC populations with similar multilineage differentiation potentials in vitro can be obtained from many non-BM tissues, including the adipose tissue [4], placenta [5], umbilical cord, and peripheral blood [6], and even from gingival tissue [7]. In the last decade, MSCs held great promise for cell-based therapy in several disorders, including immune disorders and tumors, due to their ability to home to damaged tissues, to release bioactive molecules, and to have immunomodulatory actions. Moreover, MSCs have been shown to cross the blood-brain barrier (BBB), a characteristic that represents an important aspect when considering MSCs as a therapeutic option for brain tumors. Although the mechanisms driving MSCs to their cellular targets are still widely unknown, the therapeutic use of MSCs has been studied in an impressive number of clinical trials [8]. However, the issue of whether the presence of MSCs in the tumor microenvironment and the molecular crosstalk with the resident cells may result in tumor-suppressive effects or, alternatively, may favor tumor growth is still unclear. Several hypotheses have been postulated to explain the antitumor effects of MSCs, including inhibition of proliferation-related signaling pathways, such as AKT, PI3K, and Wnt, inhibition of cell-cycle progression, downregulation of XIAP (X-linked inhibitor of apoptosis protein), and suppression of angiogenesis [9–16].

Using an orthotopic glioblastoma (GBM) model, we recently demonstrated that murine MSCs have a strong tropism towards tumor cells and, when loaded with paclitaxel (PTX), induce cytotoxic damage in the tumor cells [17]. In the present work, we addressed some therapy-relevant issues for using human MSCs (hMSCs) in GBM, that included: 1) the ability of intracerebrally injected hMSCs, either loaded with PTX or unloaded, to affect tumor growth and survival; 2) whether a hMSC-based therapy may be effective against the cancer stem cell population of GBM; and 3) the efficiency of systemically injected hMSCs to home to GBM brain xenografts.

## Methods

### Cell cultures

#### Tumor cells

All cells were cultured at 37 °C in a humidified atmosphere containing 5% carbon dioxide. U87MG GBM cells (HTB14; ATCC) were cultured in high-glucose Dulbecco’s modified Eagle’s medium (DMEM; Sigma, St. Louis, MO, USA) supplemented with 10% fetal bovine serum (FBS; Gibco, Life Technologies, Waltham, MA, USA) under standard conditions. The patient-derived glioma stem-like cell (GSC) line GSC1 that had been established in our laboratory and extensively studied in brain xenografts [18] was cultured in a serum-free medium supplemented with epidermal growth factor (EGF) and basic fibroblast growth factor (bFGF) as described previously [19].

#### Human adipose-derived and bone marrow-derived mesenchymal stromal cells (Ad-hMSCs and BM-hMSCs)

According to the policies approved by the Institutional Review Boards for Human Studies local ethical committees (IRB 48/2013, Istituto Neurologico Carlo Besta), adipose tissue samples were obtained after informed consent. Two Ad-hMSC cultures, here named Ad09 and AdFV, were established from a male (34 years old) and a female (64 years old) donor, respectively. The Ad-hMSCs were isolated following a previously described procedure [20, 21].

Briefly, 5 ml sterile peri-umbilical fat lipo-aspirates for each sample were processed. Lipoaspirates were repeatedly washed with phosphate-buffered saline (PBS) for removal of residual blood at 250 × g. After the liquid phase removal, collagenase solution (0.25% w/v; Sigma) plus 200 μl DNAse (Sigma) at 1:100 dilution were added. After enzymatic digestion overnight at 37 °C, cells were washed by centrifugation at 250 × g for 10 min. The pellets were resuspended in Iscove’s modified Dulbecco’s medium (IMDM) + 5% FBS (Gibco, Life Technologies, Monza, Italy), seeded into T25 culture flasks, and incubated at 37 °C in a humidified atmosphere containing 5% CO_2_. The following day, the medium was aspirated and replaced with IMDM + 5% FBS and 50 ng/ml bFGF (Lonza, Walkersville, MD. USA). After 5–7 days of culture, the plastic adherent cells were harvested with trypsin and processed for the removal of CD31^+^ cells using magnetic beads (Invitrogen, Italy, CELLection™ Pan Mouse IgG Kit) as previously described [22]. The remaining CD31^–^ cells, i.e., Ad-hMSCs, were expanded in IMDM + 5% FBS + bFGF and routinely passaged to 70–80% confluence. To confirm their mesenchymal phenotype, Ad-hMSC cultures were characterized by flow cytometry. Briefly, after trypsinization, cells were resuspended in PBS at a concentration of 1 × 10^5^/100 μl and incubated with 10 μl conjugated primary antibody for 30 min at 4 °C in the dark. Phycoerythrin (PE) conjugate-antibodies were used: anti-human CD90PE (Millipore, Billerica, MA, USA; working dilution 1:10), anti-human CD105PE, anti-human CD73PE, and anti-human CD44PE (BD Pharmingen San Jose, CA, USA; working dilution 1:10). Aspecific staining was determined with the appropriate isotype control. At least 20,000 events were acquired for each sample on a FACS Advantage SE (BD Bioscience, San Diego, CA, USA) flow cytometer and the acquisition analyses were performed using CellQuest software (BD Bioscience). Ad-hMSCs were tested for their capacity to differentiate into osteocytes, chondrocytes, and adipocytes according to the methods suggested by Pittenger et al. [2]. Cells were plated into 35-mm Petri dishes at a density of 10,000 cells/cm^2^ in 1 ml of culture medium per Petri dish which was replaced with specific differentiation medium after 72 h. For osteogenic differentiation, cells were cultured in DMEM/F12 + 10% FBS supplemented with 10 nM dexamethasone, 10 mM glycerol-2-phospate, and 0.3 mM l-ascorbic acid (Sigma-Aldrich). After 14 days, culture monolayers were fixed in cooled methanol and then processed for alkaline phosphatase staining. For chondrocytic differentiation, cells were cultured in hMSC chondrogenesis induction medium (Provitro, Germany) according to the micro-mass method [23] with some modifications, as reported by Pessina et al. [24]. To induce adipocytic differentiation, cells were cultured in DMEM high glucose + 10% FBS supplemented with 200 μM indomethacin (Alexis Biochemicals, USA), 0.5 mM isobutylmethylxanthine, 1 μM dexamethasone, 1 μM hydrocortisone, and 10 mg/l insulin (all from Sigma-Aldrich). After 10 days of culture, cells were fixed with 4% formalin solution and then processed for Oil red O staining (Sigma-Aldrich). BM-hMSCs were purchased from Lonza (Walkersville, MD, USA) and cultured according to the manufacturer’s instructions. Table 1 summarizes the characterization of Ad09-, AdFV-, and BM-hMSC lines.

**Table 1:** Phenotypic characterization of hMSCs

Phenotypic markers	Ad-09	Ad-FV	BM
CD90	+	+	+
CD105	+	+	+
CD73	+	+	+
CD44	+	+	+
CD166	+	+	+
CD31	–	–	Not tested
CD40	–	–	Not tested
CD45	–	–	–
CD34	–	–	–
HLA II	–	–	–
CD14	Not tested	Not tested	–
CD19	Not tested	Not tested	–

*BM* bone marrow, *hMSC* human mesenchymal stem/stromal cell

### Production of viral stocks and cell infections

All fluorescent cell strains used in our experiments were obtained by lentiviral stable transduction of either green fluorescent protein (GFP) or mCherry protein, as described in [25]. Briefly, viral particles were produced in HEK 293 T cells by transient transfection using Lipofectamine reagent (Life Technologies, Waltham, MA, USA). HEK 293 T cells were co-transfected with the lentiviral constructs pCLLsin.PPT.hPGK.GFP.pre [25] or pLVXmCherry-C1 (Clonetech Laboratories Inc., MountainView, CA, USA), together with the packaging plasmids pMDL, pRSV, and VSVG. Supernatants were collected every 24 h between 48 and 72 h after transfection and used in three successive rounds of infection in the presence of 8 μg/ml polybrene. Lentiviral infection occurred with high efficiency, as assessed by green or red fluorescence, so that no enrichment for transduced cells was required.

### Loading of Ad-hMSCs with PTX

GFP-MSCs were loaded with 2000 ng/ml PTX (2340 nM) (Adipogen, Liestal, Switzerland), as described in Pessina et al. [26]. Briefly, cells were incubated with PTX for 24 h. At the end of incubation, cells were trypsinized, extensively washed with HBSS, and seeded in a new flask. After 24 h, conditioned medium (CM) was collected and used to culture U87MG. Parallel cultures of U87MG cells were grown with CM from unloaded MSCs and used as controls.

### MTS assay

The effect of CM from PTX-hMSCs was evaluated both on U87MG and on GSC cell viability by Cell Titer 96 Aqueous One Solution Cell Proliferation Assay (Promega, Madison, WI, USA). Cells were seeded on a 96-well plate and cultured for 5 days in the presence of serial 1:2 dilutions of CM from PTX-loaded or unloaded Ad-hMSCs, or serial 1:2 dilutions of PTX (initial concentration 100 ng/ml). Cell viability was calculated as the ratio between the absorbance of treated and control (sham-treated) tumor cells. Mean values and standard deviation were generated from two biological replicates. Each experiment was performed at least three times. Representative results of a single experiment are shown in Additional file 1: Figure S1. Three independent experiments were consistent.

### Cocultures

Coculture experiments were performed by plating GFP-labeled tumor cells (U87MG or GSCs) mixed with Cherry-labeled Ad-hMSCs at a 3:1 ratio on glass slides. We performed the experiment using either PTX-loaded or unloaded Ad-hMSCs. Twenty-four or 48 h later, cells were fixed in 4% paraformaldehyde for 20 min at room temperature. Fixation was quenched by washing twice with PBS. Cells were permeabilized with PBS containing 0.3% Triton X-100 for 20 min at room temperature in a humid chamber, and then incubated in 4′,6-diamidino-2-phenylindole (DAPI; 1:4000) for 10 min and washed before mounting the coverslips with mounting medium. The cytotoxic effect induced by PTX release was evaluated by counting the tumor cells with aberrant spindles and the multinucleated tumor cells.

### Orthotopic xenografting of U87MG cells and patient-derived GSC1

Orthotopic xenograft models of human GBM cells were developed in immunosuppressed adult rats (males; 200–250 g). Either athymic nude rats (Charles River, Milan, Italy) or cyclosporine immunosuppressed rats (Wistar, Università Cattolica Breeding Laboratory, Rome, Italy) were used.

For cyclosporine immunosuppression, the rats were treated with subcutaneous injections of cyclosporine (30 mg/kg, three times per week) beginning 7 days before tumor implantation. For brain grafting, the rats were anesthetized with an intraperitoneal injection of diazepam (2 mg/100 g) followed by intramuscular injection of ketamine (4 mg/100 g). Animal skulls were immobilized in a stereotactic head frame. A burr hole was made 1 mm anterior to the bregma and 4 mm right of the midline. The tip of a 10-μl Hamilton microsyringe was placed at a depth of 5 mm from the dura and 3 × 10^5^ of either GFP-labeled U87MG or GSC1 were injected over 10 min.

In the U87MG xenografts, either PTX-loaded or unloaded hMSCs were injected immediately after tumor cell implantation through a second hole that was made 1 mm anterior to the bregma and 2 mm right of the midline. Fluorescently labeled Ad-hMSCs (10^5^ cells) were injected at 5 mm depth from the dura. In the GSC1 xenografts, injection of Ad-hMSCs (either PTX-loaded or unloaded) was made 16 weeks after tumor cell implantation to the same stereotactic coordinates as used for tumor grafting. Control rats were grafted with 3 × 10^5^ of either GFP-U87MG or GFP-GSC1 cells or saline. After surgery, the animals were kept under pathogen-free conditions in positive-pressure cabinets (Tecniplast Gazzada, Varese, Italy) and observed daily for neurological signs and body weight loss.

### Systemic injection of hMSCs

Systemic injection of either Ad- or BM-hMSCs was performed in athymic rats bearing U87MG xenografts by 2 weeks after U87MG cell implantation. Three routes of systemic injection of hMSCs were used: the femoral vein, the common carotid artery, and the common carotid artery after ligation of the external carotid artery. Each animal received 2 × 10^5^ fluorescent hMSCs resuspended in 300 μl saline that were slowly injected over 10 min.

### Fluorescence microscopy and immunofluorescence of brain tumor xenografts

Rats grafted with U87MG and GSC1 cells were sacrificed 1 week and 3 weeks after intracerebral hMSC injections, respectively. Rats receiving systemic hMSCs were allowed to survive from 1 to 72 h after injection.

The rats were deeply anesthetized and transcardially perfused with 0.1 M PBS (pH 7.4), followed by 4% paraformaldehyde in 0.1 M PBS. The brains were removed, post-fixed in 4% paraformaldehyde in PBS for 3 days, and cryoprotected in PBS with 30% sucrose for 3 days. Coronal sections of the brain (40-μm thick) were blocked in PBS with 10% bovine serum albumin (BSA) and 0.3% Triton X-100 for 45 min. Sections were incubated overnight at 4 °C with primary antibodies in PBS with 0.3% Triton X-100 and 0.1% normal donkey serum (NDS). Monoclonal antibodies used were: anti-rabbit Ki-67 (1:150; Thermo Fisher Scientific, Waltham, MA, USA) and mouse anti-Rat Blood-Brain Barrier (Clone SMI-71) (1:500; Biolegend, San Diego, CA, USA). Polyclonal antibodies used were: rabbit anti-Iba1 (1:200; Wako Chemicals, Richmond, VA, USA), goat anti-CD34 (C-18) (1:50; Santa Cruz Biotechnology, Dallas, TX, USA), rat anti-mouse CD31 (1:100; BD Bioscience, Franklin Lakes, NJ, USA), and rabbit anti-GFAP (1:1000; Dako Italia, Milan, Italy). For detecting brain microvessels, sections were incubated overnight at 4 °C in PBS with 0.3% Triton X-100 and 0.1% NDS with lectin from *Lycopersicon esculentum* (tomato) biotin conjugate (1:500; Sigma-Aldrich, St. Louis, MO, USA) together with primary antibodies. Slices were rinsed and incubated in PBS containing 0.3% Triton X-100 with secondary antibodies for 2 h at room temperature. Secondary antibodies used were: Alexa Fluor 647 or 555 or 488 donkey anti-mouse, Alexa Fluor 488 or 555 or 647, donkey anti-rabbit secondary antibodies (1:500; Thermo Fisher Scientific, Waltham, MA, USA), Alexa Fluor 488 or 555 donkey anti-goat antibodies (1:400; Thermo Fisher Scientific, Waltham, MA, USA), and Cy3 donkey anti-Rat (1:200; EMD Millipore, Billerica, MA, USA).

For lectin immunostaining, sections were incubated for 2 h at room temperature in PBS containing 0.3% Triton X-100 with streptavidin protein, DyLight 405 conjugate, or streptavidin Alexa Fluor® 647 conjugate (1:200; Thermo Fisher Scientific, Waltham, MA, USA). Before mounting, slices were incubated with DAPI (1:4000; Sigma-Aldrich) for 10 min. Immunofluorescence was observed with a laser confocal microscope (SP5; Leica) and images were acquired. Image analysis was performed with Leica Application Suite X software.

### Tumor volume calculation

For each brain, serial thick sections (40 μm) starting from the olfactory bulb to the cerebellum were prepared. Slices were collected in a 24 multiwell plate. Every brain slice was consecutively placed in a well starting with well 1 and ending with well 24. This procedure was repeated until the whole brain was cut. For brain tumor volume calculation, the brain slices of one tube were transferred to a new well and incubated for 20 min in PBS containing 0.3% Triton X-100 with DAPI (1:4000; Sigma-Aldrich). Fluorescent tumor area of every slice was observed and acquired with a laser confocal microscope (SP5; Leica). Images were computer processed in ImageJ (National Institutes of Health) in order to determine the tumor areas of each slice in a well. The following formula was used to calculate brain tumor volume: tumor volume = slice size (40 μm) × step size between the slices (24) × sum of tumor areas from one well. The tumor volume for each brain was calculated by analyzing slices from at least two wells.

### Assessment of microvascular density

For angiogenesis quantification we determined the density of tumor vessels stained by immunoreaction with the anti-CD34 antibody. We acquired at least 20 no-overlapping images (20× magnification) of each tumor. Microvascular density was assessed by evaluating the fraction of tumor area occupied by CD34-positive endothelial cells per microscope field using NIH ImageJ software for image processing (https://imagej.nih.gov/ij/).

### Statistical analysis

Results are presented as mean ± standard error of the mean (SEM) and statistically evaluated by a two-tailed Student’s *t* test. Data from cocultures are evaluated by one-way analysis of variance (ANOVA). Multiple comparisons were performed using the post-hoc Bonferroni comparison test. Survival curves were plotted using the Kaplan-Meier method and differences between groups were evaluated using the log-rank test. Statistical significance was assigned to *p* values <0.05. All statistical analyses were performed with GraphPad Prism 5 software (GraphPad Software, San Diego, CA, USA).

## Results

### Intracerebral injections of Ad-hMSCs in U87MG orthotopic xenografts

In a previous study, we showed that murine PTX-loaded MSCs injected adjacently to U87MG brain xenografts are able to migrate towards the tumor and to induce PTX-specific cytotoxic damage in the tumor cells [17]. The present experiment was designed to investigate whether Ad-hMSCs may exert an antitumor effect comparable to that of murine MSCs, and if such an effect may be relevant to animal survival. Both unloaded and PTX-loaded Ad-hMSCs were assessed for their ability to migrate towards the tumor and to exert antiproliferative effects. To preliminary characterize in vitro the efficiency of PTX loading/releasing by Ad-hMSCs, we followed our standardized procedure [26]. The ability of CM from PTX-loaded Ad-hMSCs to affect U87MG cell viability was addressed by MTS assay, in comparison with U87MG cells treated with scaled doses of PTX. CM from unloaded Ad-hMSCs was used as the negative control. Ad-hMSCs showed an uptake/release capacity comparable to that of murine MSCs [17, 26] (Additional file 1: Figure S1). Moreover, we cocultured U87MG cells either with PTX-loaded or with unloaded Ad-hMSCs at a 3:1 ratio. After 24 or 48 h, cells were fixed and analyzed with respect to their spindle morphology and nuclear content (Additional file 2: Figure S2a). At 24 h of coculturing, the percentage of U87MG with mono/multipolar spindles was significantly higher in cocultures with PTX-Ad-hMSCs as compared to U87MG cocultured with unloaded Ad-hMSCs (Additional file 2: Figure S2b) or to single U87MG cell cultures (data not shown). At 48 h of coculturing, we observed an impressive increase in multinucleated U87MG tumor cells in coculture with PTX-Ad-hMSCs as compared to U87MG cells cocultured with unloaded Ad-hMSCs (Additional file 2: Figure S2c) or to single populations of control U87MG (data not shown).

**Fig. 1:**
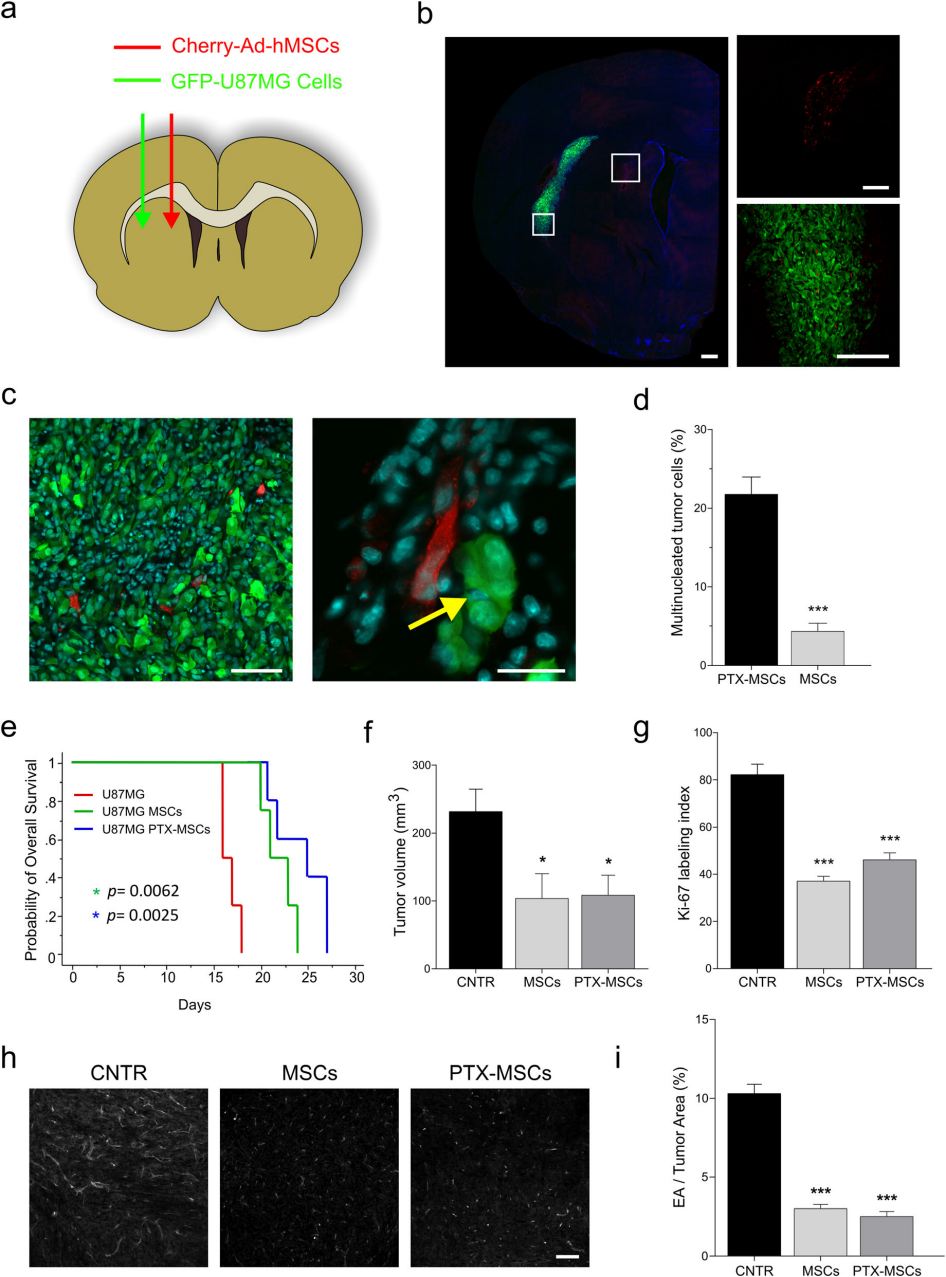
Intracerebral injections of hMSCs in U87MG orthotopic xenografts. **a** Schematic drawing of the intracerebral injection of m-Cherry adipose-derived human mesenchymal stem/stromal cells (*Ad-hMSCs*) (*red*) and green fluorescent protein (*GFP*) U87MG cells (*green*) in cyclosporine immunosuppressed rats. **b** Microphotographs on fluorescence microscopy of a coronal section of rat brain showing the injection sites at low (*left panel*; *scale bar* = 500 μm) and higher (*right panel*; *scale bars* = 250 μm) magnification. At 1 week after grafting, the vast majority of the Ad-hMSCs had migrated towards the tumor. **c** Microphotograph of the tumor epicentre showing Ad-hMSCs that have migrated into the tumor (*left panel*; *scale bar* = 40 μm). Detail of a paclitaxel (*PTX*)-loaded Ad-hMSC lying adjacent to a multinucleated U87MG tumor cell (*yellow arrow*; *scale bar* = 25 μm)*.* **d** Bar graph showing the percentage of multinucleated U87MG cells in brain xenografts injected with PTX-loaded Ad-hMSCs and in those injected with unloaded Ad-hMSCs (***p < 0.0001). **e** Kaplan-Meier curves for the survival of rats grafted with U87MG cells (*red line*), U87MG cells plus Ad-hMSCs (*green line*), and U87MG cells plus PTX-loaded Ad-hMSCs (*blue line*). **f** Bar graph showing the volume of U87MG xenografts in control rats and in rats treated with unloaded Ad-hMSCs and in those treated with PTX-loaded Ad-hMSCs (*p < 0.05). **g** Bar graph showing cell proliferation, as assessed by Ki67 labeling, in control U87MG xenografts and in those injected with unloaded Ad-hMSCs or with PTX-loaded Ad-hMSCs (***p < 0.0001). **h** Assessment of microvascular density. Sections of the brain xenograft immunostained with anti-CD34 were acquired, converted into grayscale, and assessed by computerized image analysis. *Scale bar* = 100 μm. **i** Bar graph showing microvascular density in control U87MG xenografts and in xenografts injected with unloaded Ad-hMSCs or with PTX-loaded Ad-hMSCs. Values on the *y* axis represent the percent of the endothelial area (CD34^+^)/tumor area (*EA/tumor area*) per microscope field (***p < 0.0001). *CNTR* control

Cyclosporine immunosuppressed rats were stereotactically grafted into the right striatum with GFP-labeled U87MG cells. In the same procedure, mCherry-labeled Ad09-hMSCs were injected adjacent to U87MG cells (Fig. 1a). Either PTX-loaded or unloaded Ad-hMSCs were injected in separate groups of rats. One week after grafting, the vast majority of Ad-hMSCs had migrated from the grafting site towards the tumor cells (Fig. 1b). Migration of U87MG cells towards the site of Ad-hMSC injection was also noted; however, this involved only rare tumor cells. Migrating Ad-hMSCs populated all regions of the tumor xenograft without any specific tropism, such as, for example, for the vascular structures. There were no differences in the pattern of migration through the brain parenchyma between PTX-loaded and unloaded Ad-hMSCs. Importantly, the U87MG cells that surrounded PTX-loaded Ad-hMSCs showed the typical cytotoxic effects produced by PTX, such as aberrant mitoses with multipolar spindles, eventually resulting in multinucleated tumor cells (Fig. 1c). By analyzing the area within a radius of 50 μm from each Ad-hMSC, we found that the percentage of multinucleated cells was 21.68 ± 2.22% (mean ± SEM) and 4.27 ± 1.03% in the U87MG xenografts injected with PTX-loaded Ad-hMSCs and unloaded Ad-hMSCs, respectively (*p* < 0.0001 by Student’s *t* test) (Fig. 1d). At distances further than 50 μm from any single Ad-hMSC, the fraction of multinucleated U87MG cells dropped massively.

To assess whether intracerebral Ad-hMSC injections may have any effect on survival, we grafted the GFP-labeled U87MG cells onto the striatum of immunosuppressed rats and treated the rats with a single intracerebral injection of either mCherry Ad-hMSCs (*n* = 4) or PTX-loaded mCherry Ad-hMSCs (*n* = 5), or saline (*n* = 4). The animals were sacrificed after the appearance of neurological deficits or by losing >20% of their body weight. Kaplan-Meier analysis showed that rats injected with Ad-hMSCs survived significantly longer than controls (Fig. 1e). Although the median survival was better in rats treated with PTX-loaded Ad-hMSCs, there was no significant difference in survival between the animals injected with PTX-loaded Ad-hMSCs and those injected with unloaded Ad-hMSCs (median survival 24 and 21 days in PTX-loaded and unloaded Ad-hMSCs, respectively; *p* = 0.2404 by Student’s *t* test). In order to relate survival to tumor growth and pathology, a subset of rats was sacrificed at 2 weeks after grafting and the brain was assessed for tumor volume, tumor cell proliferation, and microvascular density (Fig. 1f–i). Both rats treated with unloaded Ad-hMSCs (*n* = 3) and those treated with PTX-loaded Ad-hMSCs (*n* = 3) harbored significantly smaller brain tumors than saline-treated controls (*n* = 3) (Fig. 1f). The Ki-67 labeling index was significantly reduced both in tumors treated with unloaded Ad-hMSCs and in those treated with PTX-loaded Ad-hMSCs (Fig. 1g). Cell proliferation was 82.04 ± 4.48% (mean ± SEM), 37.00 ± 2.06%, and 46.05 ± 2.97% in xenografts injected with U87MG cells plus saline, or with U87MG plus unloaded Ad-hMSCs, or with U87MG plus PTX-loaded Ad-hMSCs, respectively (*p* < 0.0001 both for U87MG vs U87MG plus hMSCs and for U87MG vs U87MG plus PTX-loaded hMSCs by Student’s *t* test).

The microvascular density was significantly reduced compared with saline-injected controls for both the U87MG xenografts treated with unloaded and in those treated with PTX-loaded Ad-hMSCs. Microvascular density scored 10.27 ± 0.59 (mean ± SEM), 2.98 ± 0.26, and 2.48 ± 0.32 in control U87MG, U87MG plus Ad-hMSCs, and U87MG plus PTX-loaded Ad-hMSCs, respectively (*p* < 0.0001 by Student’s *t* test).

Overall, these experiments demonstrated that intracerebral injections of Ad-hMSCs have a therapeutic potential in U87MG brain xenografts by prolonging animal survival. This potential is related to inhibited tumor growth due to decreased cell proliferation and tumor angiogenesis. In this context, PTX loading of Ad-hMSCs seems to play a minor role. Although injection of PTX Ad- hMSCs yielded the best results in terms of survival and PTX-induced damage could be detected in the tumor cells, PTX did not add significant advantages to the inhibition of tumor growth, cell proliferation, or angiogenesis by the Ad-hMSCs.

### Intratumoral injections of Ad-hMSCs in established orthotopic xenografts of patient-derived GSCs

GSCs have been shown to generate tumor xenografts that reproduce the parent tumor more closely than serum-cultured GBM cell lines, such as the U87MG cells [19]. Furthermore, GSCs are highly resistant to anticancer drugs [27, 28] and are believed to be responsible for tumor recurrence after surgery and radiochemotherapy. From a translational point of view, it is important to know whether locally applied hMSCs may be effective against GSCs; for example, to sterilize the tumor bed after surgical resection. We aimed to investigate the therapeutic potential of intratumoral hMSC injections in established brain xenografts of patient-derived GSCs. To preliminary test the effects exerted on GSCs by exposure to Ad-hMSCs, we applied the same approach used for U87MG. We either exposed GSCs to the CM from PTX-loaded or unloaded Ad-hMSCs or, alternatively, we cocultured GSCs with PTX-loaded or unloaded Ad-hMSCs (3:1 ratio). As expected, PTX cytotoxicity was lower than that observed on U87MG cells. However, CM from PTX-loaded Ad-hMSCs was able to induce a cytotoxic effect on GSCs as well (Additional file 1: Figure S1). Moreover, when we cocultured GSCs with PTX-Ad-hMSCs for 24 h (Additional file 2: Figure S2a) we found that the aberrant spindles were significantly increased in the tumor cell nuclei (Additional file 2: Figure S2b). After 48 h, there was a significant increase in multinucleated GSCs in cocultures with PTX-Ad-hMSCs (Additional file 2: Figure S2c). No significant difference was observed in control cocultures (GSCs/Ad-MSCs) (Additional file 2: Figure S2c) or in single GSC cell cultures (data not shown).

**Fig. 2:**
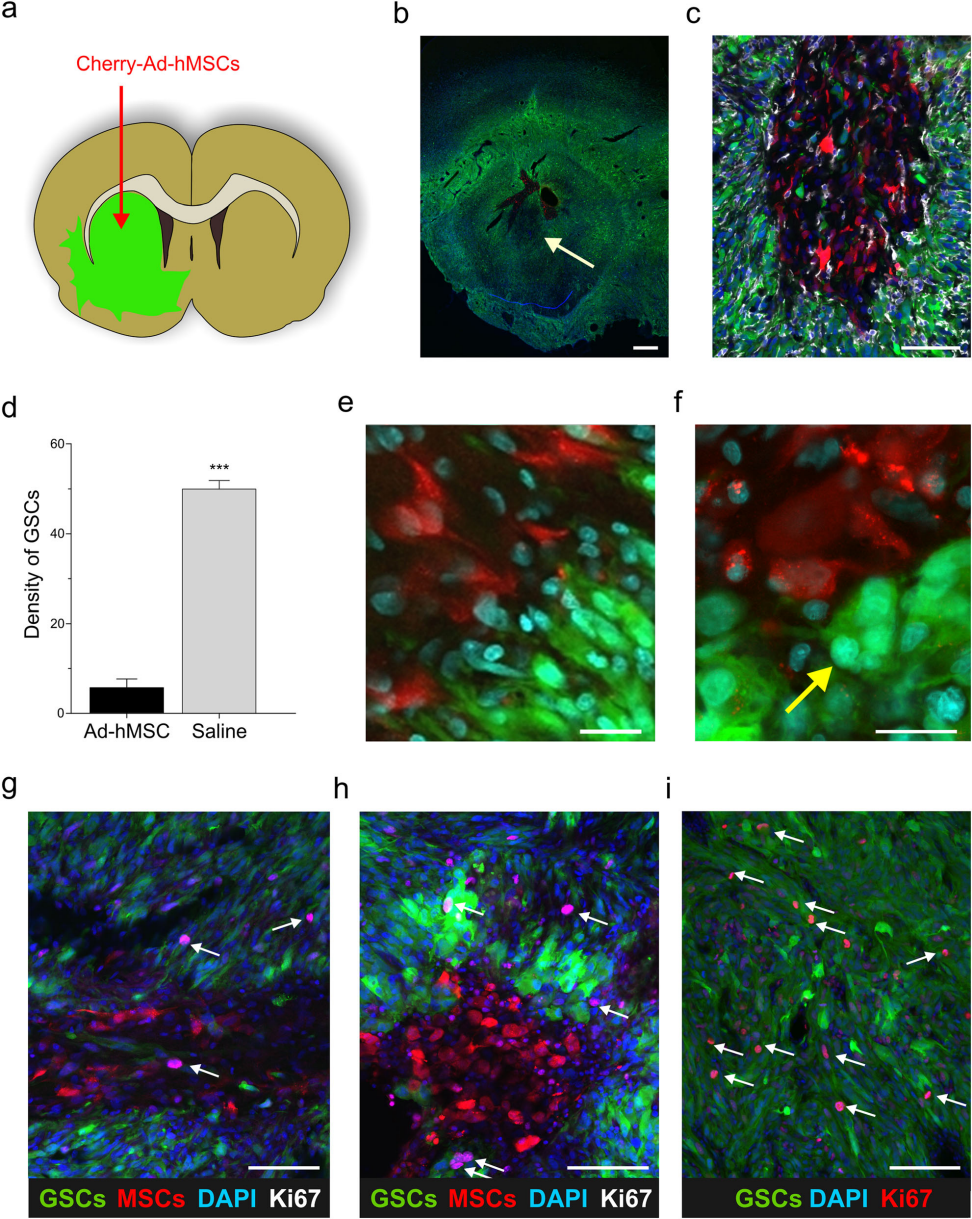
Intratumoral injections of hMSCs in orthotopic xenografts of glioma stem-like cells (*GSCs*). **a** Schematic drawing of the intratumoral injection of m-Cherry adipose-derived human mesenchymal stem/stromal cells (*Ad-hMSCs*) (*red*) in athymic rats with established brain xenografts of patient-derived GSC1 tumor cells (*green*). **b** Low-power fluorescence microphotograph of a coronal section of rat brain through the tumor epicentre showing the injection sites of Ad-hMSCs (*arrow*). *Scale bar* = 500 μm. **c** Microphtograph of GSC1 xenograft immunostained with anti-Iba1 (*white staining*) showing an area injected with Ad-hMSCs with depletion of tumor cells and microglial reaction. *Scale bar* = 75 μm. **d** Bar graph showing the mean density of GSC1 tumor cells in regions injected with Ad-hMSCs (*grey bar*) and in saline-injected region of control tumors (*black bar*; ***p < 0.0001). **e** Detail of interaction between unloaded Ad-hMSCs and GSC1 tumor cells*. Scale bar* = 25 μm. **f** Detail of PTX-loaded Ad-hMSCs lying adjacent to a multinucleated GSC1 tumor cell (*yellow arrow). Scale bar* = 25 μm. **g**–**i** Microphotographs showing cell proliferation, as assessed by Ki67 labeling, in GSC1 xenografts injected with **g** unloaded Ad-hMSCs, **h** with PTX-loaded Ad-hMSCs, and **i** with saline. The *arrows* point out proliferating tumor cells. *Scale bars* = 75 μm

GFP-expressing GSC1 cells were grafted onto the striatum of athymic rats (*n* = 11). Sixteen weeks later, mCherry-Ad-hMSCs were injected intracerebrally at the same stereotactic coordinates used for tumor cell grafting (Fig. 2a). For this series of experiments, we used the mCherry FVAd-hMSCs, which were injected either loaded with PTX (*n* = 4 rats) or unloaded (*n* = 4 rats) (Additional file 1: Figure S1). Three weeks after the injection of Ad-hMSCs, these cells were found to populate the tumor core (Fig. 2b). Interestingly, the density of GSC1 cells in areas injected with Ad-hMSCs was much lower than in the injected regions of saline-treated xenografts (*n* = 3 rats). In regions injected with Ad-hMSCs, immunostaining with anti-Iba1 showed a strong microglial activation that was associated with depletion of tumor cells, suggesting an inflammatory reaction with phagocytic activity (Fig. 2c). Overall, the tumor regions injected with Ad-hMSCs showed GSC density of 5.64 ± 1.94 (mean ± SEM) cells/100 μm^2^, whereas GSC density in saline-injected regions of control xenografts was 49.9 ± 1.91 cells/100 μm^2^ (*p* < 0.0001 by Student’s *t* test) (Fig. 2d). There were no differences in GSC density between tumor regions injected with PTX-loaded Ad-MSCs and those injected with unloaded Ad-hMSCs. However, engraftment of PTX-loaded Ad-hMSCs resulted in multinucleated tumor cells (Fig. 2e and f). As compared with saline-injected controls, proliferation of GSCs was significantly reduced both in xenografts injected with unloaded Ad-hMSCs and in those injected with PTX-loaded Ad-hMSCs (Fig. 2g–i). The Ki-67 labeling index was 14.35 ± 2.67% (mean ± SEM), 10.75 ± 1.25%, and 23.5 ± 2.38% in xenografts injected with Ad-hMSCs, PTX-loaded Ad-hMSCs, and saline, respectively (Ad-hMSCs vs control, *p* = 0.0437; PTX-loaded Ad-hMSCs vs control, *p* = 0.0068; Ad-hMSCs vs PTX-loaded Ad-hMSCs, *p* = not significant; all by Student’s *t* test).

Together, the experiments on intracerebral injection of Ad-hMSCs demonstrated that local therapy with hMSCs may be an effective strategy not only against the bulk of GBM, modeled by the U87MG cell xenografts, but also against GSCs, which represents the subpopulation of tumor cells responsible for resistance to radiochemotherapy and recurrence.

### Systemic injection of hMSCs in established orthotopic xenografts of U87MG cells

**Fig. 3:**
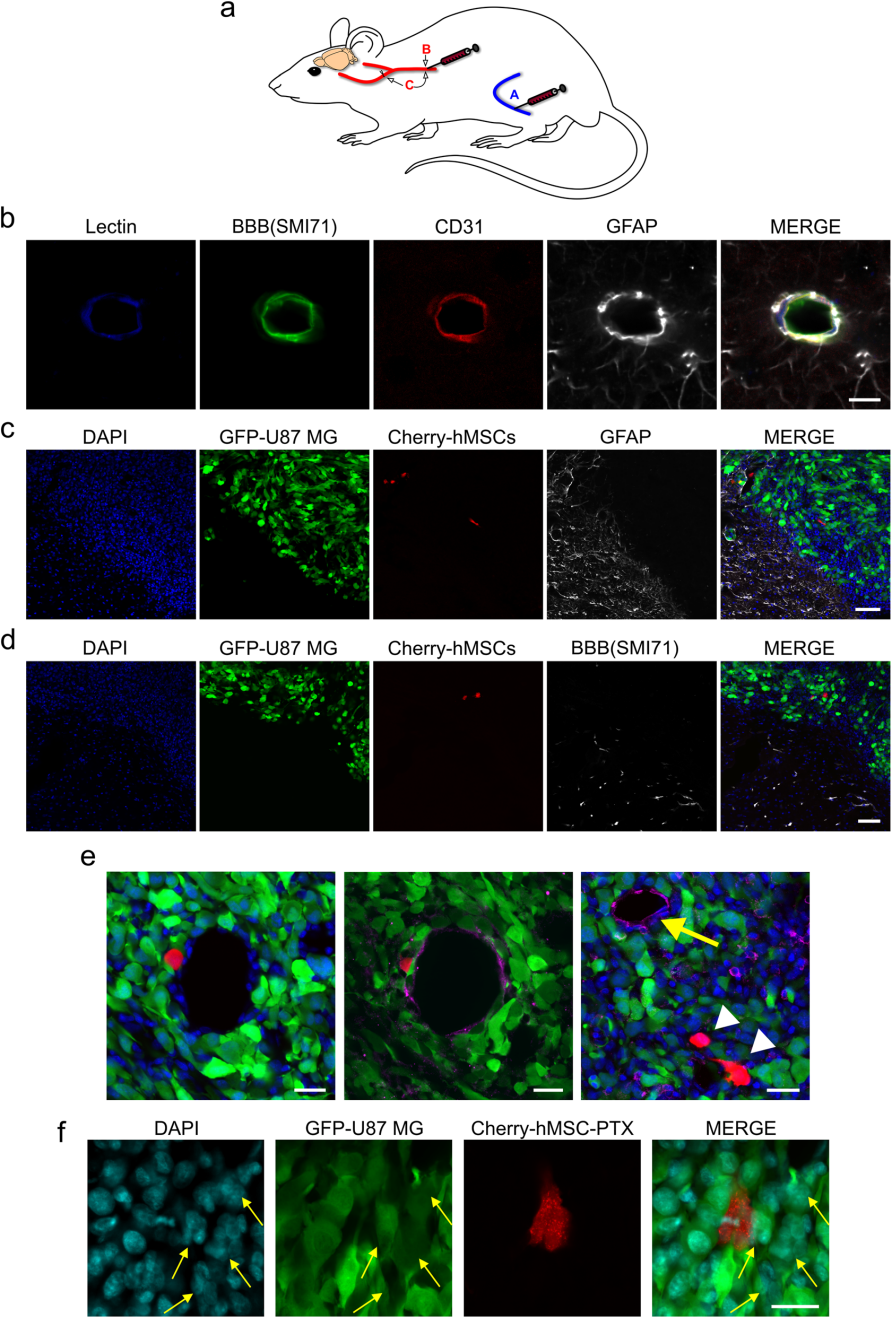
Systemic injection of hMSCs in established orthotopic xenografts of U87MG cells. **a** Schematic drawing of systemic injections of m-Cherry hMSCs in athymic rats with established brain xenografts of U87MG cells. The hMSCs were injected either into the femoral vein (A), or into the common carotid artery (B), or into the common carotid artery after ligation of the external carotid artery (C). Intra-arterial injections were performed on the same side of the brain xenograft. **b** A microvessel lying in the brain hemisphere contralateral to the tumor that was immunostained with antibodies for the endothelium (lectin, CD31), glial cells (GFAP), and blood-brain barrier (*BBB*) (SMI71). *Scale bar* = 10 μm. **c**,**d** Microphtographs of the brain-tumor interface after intravenous injection of m-Cherry adipose-derived human mesenchymal stem/stromal cells (*Ad-hMSCs*). Intratumoral Ad-hMSCs were found only in regions with absent perivascular astrocytic endings (**c**) and SMI71 immunostaining (**d**). *Scale bar* = 75 μm. **e** Microphtographs of tumor microvessels obtained 1 h after intracarotid injection of m-Cherry BM-hMSCs showing transmigration through the vessel wall (*center*, anti-CD31 in *purple*; *right*, lectin in *purple*). *Scale bar* = 25 μm. **f** Detail of a brain xenograft after intracarotid injection of paclitaxel (*PTX*)-loaded Ad-hMSCs. One Ad-hMSC is surrounded by three multinucleated U87MG tumor cells (*yellow arrows*)*. Scale bar* = 25 μm

On a translational basis, direct injection of hMSCs into the brain parenchyma may reasonably be proposed at primary surgery to sterilize the resection cavity, but it is challenging in cases of tumor recurrence after standard radiochemotherapy due to the iatrogenic risks of multiple intracerebral injections along the paths of the recurrent tumor. With regard to a less invasive approach to tumor recurrences, we investigated the ability of systemically injected hMSCs to home to the parenchyma of GBM through the tumor vasculature and the potential of these cells to exert any anticancer activity *per se* or after PTX loading. We first established brain tumor xenografts in athymic rats using GFP-labeled U87MG cells (*n* = 19). Two weeks later, we injected either mCherry-AdFV-hMSCs (*n* = 9) or mCherry-BM-hMSCs (*n* = 8) or saline (*n* = 2) using different routes in separate rats, i.e., the femoral vein, the common carotid artery, and the common carotid artery after ligation of the external carotid artery (Fig. 3a). The xenografts were assessed by confocal microscopy from 1 h to 3 days after hMSC injection. Results showed that systemically injected hMSCs are able to home to GBM parenchyma through the tumor vasculature (Fig. 3b–e). This process takes place as early as 1 h after injection and occurs mostly at the tumor periphery, where angiogenesis is highly remarkable (Fig. 3c). Strikingly, hMSCs were able to cross the vessel wall only in regions where the architecture of the brain vascularity was disrupted, as demonstrated by immunofluorescence with markers for glial and endothelial cells, and for the BBB (Fig. 3d). In regions where the brain vessels maintained their normal architecture, extravasation of hMSCs did not occur. Overall, the efficiency of hMSCs homing to tumor xenografts was low and related both to the route of cell injection and to the source from which the hMSCs were derived. After injection into the femoral vein, about 0.02% of Ad-hMSCs engrafted at the site of the brain tumor. Conversely, injection of BM-hMSCs into the common carotid artery homolaterally to the tumor yielded an engraftment rate of 0.1%, which increased to 0.5% by selectively injecting into the internal carotid artery. Interestingly, the amount of hMSCs that were found in the lung, which works as a filter organ for circulating hMSCs, decreased from 1 h to 24 h after systemic injection, whereas the number of hMSCs homing to the brain xenograft increased over the same time frame, suggesting their ability to mobilize to sites of vascularized tumor tissue (Additional file 3: Figure S3). Systemically injected PTX-loaded Ad-hMSCs maintained their ability to home to the brain tumor and induced cytotoxic damage to the surrounding tumor cells (Fig. 3f).

Of note, there was no mortality/morbidity related to the injection of hMSCs, both after intravenous or intra-arterial administration. On histological examination of the lungs there were no findings suggesting pulmonary embolism.

## Discussion

MSCs have gained considerable attention as a valuable tool for cell therapy due to their intrinsic peculiarities; first of all, their innate tropism for sites of injury, either of inflammatory or neoplastic nature. MSCs are taken into consideration as potential antitumor cells as well as vehicles for gene therapy or for drug delivery. However, the key issue of the effect the MSCs may exert on tumor growth is still unclear. MSCs can indeed recruit more immune cells into the tumor microenvironment, increase the proportion of cancer stem cells, and promote tumor angiogenesis, further supporting tumor progression. On the other hand, as plasticity is a fundamental feature of MSCs, they can also inhibit tumor development by activating different signaling pathways, eventually resulting in an inhibitory effect on tumor growth. Such a dual role of MSCs on cancer biology has been widely addressed in literature [29], where both protumorigenic [30, 31] and counter-tumorigenic effects are reported [9–16, 32]. These discrepancies might depend on a wide range of variables, including differences in experimental settings, heterogeneity of MSCs, dose or timing of MSC injection, and animal host or tumor models. For instance, the dose of MSCs delivered is a critical factor influencing tumor growth [33]. The timing at which MSCs are inoculated into the tumor environment is another important issue. The presence of MSCs during tumor development, as in the case of co-injection of tumor cells and MSCs, undoubtedly influences the tumor environment interfering with processes such as angiogenesis and inflammation, which have a strong impact on tumor growth. Interestingly, an opposite effect of hMSC transplantation in a 4 T1 breast adenocarcinoma model has been reported [34]. In this study, injection of hMSCs derived from a single donor and grown in vitro under the same conditions in animals with the same disease resulted in a tumor-inhibiting or tumor-promoting effect depending on the experimental protocol applied (co-injection of hMSCs and tumor cells or injection of hMSCs in tumor-bearing mice). Finally, MSCs may impact on the immunosuppressive mechanisms in experimental mice models. In the central nervous system (CNS) of experimental autoimmune encephalomyelitis mice treated with MSCs, a significant reduction of inflammation has been reported, with a potent immunomodulatory effect able to reduce neuroinflammation [35].

Opposite effects in in-vitro versus in-vivo models have also been reported [36]. Moreover, the dichotomous effect of MSCs has also been described in tumor angiogenesis [16, 37–43]. The secretome of MSCs contains a plethora of angiogenic mediators [44], whose ultimate effect on tumor angiogenesis likely depends on the interaction between MSCs and the tumor microenvironment.

We previously demonstrated that murine BM-MSCs are able to inhibit tumor growth in subcutaneous grafts of U87MG cells, and that this effect is enhanced by loading the MSCs with PTX [26]. The effects exerted by PTX treatment on MSCs, both in terms of cell proliferation and differentiation ability, have been addressed by several recent papers. Ad-MSCs exposed to PTX lose their multipotent differentiation capability both in in-vitro human Ad-MSC populations and ex-vivo in rat Ad-MSC populations [45]. When PTX-treated hMSCs are induced to undergo adipogenesis they exhibit a 40% reduction in lipid accumulation and adopt fibroblast-like characteristics, including upregulation of fibroblast markers and reduction of stemness markers [46]. Similarly, PTX treatment strongly impairs differentiation of AD-hMSCs towards the adipogenic, osteogenic, and endothelial phenotypes [47].

We also showed that orthotopically injected murine BM-MSCs loaded with PTX migrate towards U87MG brain xenografts and induce cytotoxic damage in the tumor cells [17]. One point we addressed here was using Ad-hMSCs in orthotopic GBM models. The adipose tissue is less invasive and less expensive than BM as a source of hMSCs. In addition, Ad-hMSCs remain free of oncogenic transformation for at least 8 months after injection in immunocompromised mice, demonstrating more oncogenic resistance than BM-MSCs [48].

There are several strengths in the methods of the present study. One concerns the use of hMSCs that constitutively express the fluorescent mCherry protein. Although packing of the mCherry protein in the cytoplasm may alter vitality and/or motility of hMSCs, constitutive labeling of hMSCs avoids the risk of false-positive results due to spurious labeling which are inherent with the use of cell tracers [49]. Another relevant aspect of our methods is the intratumoral injection of hMSCs in the GSC xenograft, an experimental condition where the therapeutic potential of hMSCs is tested against the radiochemoresistant tumor cell population. Finally, planning different routes for systemic administration provided valuable information on homing efficiency of hMSCs to brain tumors.

Results from intracerebral injections in U87MG xenografts showed that the Ad-hMSCs are able to migrate through the brain and to colonize the tumor, eventually leading to a significant extension of survival that was associated with reduction of cell proliferation and neo-vessel formation.

Although an anti-angiogenic effect of hMSCs has previously been reported in different tumor models [16, 37, 39], the effect of Ad-hMSCs on microvessel density in our U87MG xenografts was so striking as to deserve further investigation. Current hypotheses that have been postulated to explain the inhibition of tumor angiogenesis by hMSCs include promotion of endothelial cell apoptosis [50], modulation of the VE-cadherin/beta-catenin pathway [39, 40], and suppression of vascular endothelial growth factor (VEGF) expression [43]. In a glioma model, MSCs have been demonstrated to exert anti-angiogenic activity through downregulation of the platelet derived growth factor (PDGF)-PDGFR axis, which is known to play a key role in glioma angiogenesis [38].

The rationale for using GSCs in our study stems from the notion that these cells give rise to tumor xenografts closely mimicking the histological features of human GBM and that they are responsible for therapy resistance and recurrence, whereas the serum-cultured GBM cells, such as the U87MG line, may be assimilated to the tumor bulk. One major result of our study is that the inhibitory action of hMSCs is exerted both on the proliferating cells of the tumor bulk and on the population of cancer stem cells (CSCs) responsible for relapse and therapy resistance. A recent study produced similar results, demonstrating that Ad-hMSCs target brain tumor-initiating cells isolated from the medulloblastoma, atypical teratoid/rhabdoid tumors, and GBM [51]. Moreover, Ad-hMSCs engineered to produce BMP4 effectively target CSC-derived tumors in a rat model, resulting in extended animal survival [52].

Unexpectedly, PTX loading of hMSCs did not substantially affect the growth of U87MG xenografts. We noted only a modest increase in the median survival in U87MG xenografts (3/21 days, 17%) and some reduction of cell proliferation in GSC1 xenografts (10.75 *vs* 14.35%, –25%), both of which were not significant on statistical analysis. In spite of the detection of PTX-induced cytotoxicity both in U87MG and in GSC1 xenografts, overall tumor growth was not significantly inhibited by the PTX-loaded Ad-hMSCs compared with the unloaded Ad-hMSCs. One explanation may be that PTX, although providing the Ad-hMSCs with a powerful cytotoxic agent against the tumor cells, may alter synthesis and/or secretion of those factors involved in the antitumor action of Ad-hMSCs. Alternatively, the brain region surrounding PTX-loaded Ad-hMSCs where the released drug reaches cytotoxic levels may be too small to affect the overall tumor growth. Actually, multinucleated tumor cells were mainly found within a radius of 50 μm around the PTX-loaded hMSCs and their number did not exceed five tumor cells per single Ad-hMSC.

Another key aspect of our study concerns the potential of using systemically administered hMSCs for tumor therapy. This issue has recently been addressed by other groups with encouraging results; however, some crucial points, such as hMSCs crossing the brain endothelium and drug-loaded hMSCs homing to the tumor, have not been investigated [53, 54]. We here demonstrate that hMSCs, even when loaded with PTX, are able to extravasate and to localize in the brain tumor. Importantly, this process takes place only where the BBB is disrupted, whereas in regions where the brain vessels maintained their normal architecture extravasation of hMSCs did not occur. Although our data provide evidence that hMSCs can be systemically administered as an innovative therapeutic option for GBM, the low homing efficiency of these cells to brain xenografts may limit the feasibility of this approach. Low homing efficiency of hMSCs in tumors has also been reported by other researchers, who attempted to increase homing by modulating the expression of surface ligands that bind to intercellular adhesion molecules or by preconditioning in vitro the hMSCs with GBM conditioned medium and with matrix proteins [53, 55]. For example, Smith et al. [53] reported that approximately 0.1% of Ad-hMSCs injected via the intracardiac route homed to U87MG brain xenografts and that less than one-half of this amount reached the tumor from injections into the tail vein. For both delivery routes, prime pretreatment resulted in a sixfold increase in the engraftment rate. In an ongoing study, we have found that preconditioning hMSCs in vitro with tumor necrosis factor (TNF)-α increases their adhesion to brain microvascular endothelial cells, suggesting increased homing efficiency in vivo. To enhance homing to GBM, however, we prefer to exploit selective modalities for cell injection rather than ex vivo manipulating the hMSCs, a procedure that is likely to complicate the clinical use of these cells. Actually, selective injection of hMSCs into the internal carotid artery homolaterally to the brain xenograft was fivefold more efficient than the intracardiac route and gave homing rates quite similar to those achieved by the intracardiac administration of primed hMSCs. From a clinical perspective, intra-arterial injections of hMSCs can be performed in a highly selective fashion and, above all, can be safely repeated as in the multistage embolization of cerebral arteriovenous malformations.

## Conclusion

The present work demonstrates that hMSCs are able to migrate to and colonize GBM tumor xenografts when injected directly into the brain or when systemically administered. The interaction of hMSCs with the tumor microenvironment results in a significant extension of animal survival, reduced tumor volume, and impaired cell proliferation and vascularization. Although further studies are necessary to characterize this interaction at the molecular level, our data support the possibility of clinical use of hMSCs for treating malignant gliomas.

## Additional files

Additional file 1: Figure S1. In vitro U87MG and GSC cell viability after treatment with CM from PTX-loaded Ad-hMSCs. The kinetic of growth inhibition induced by serial 1:2 dilutions of CM from PTX-loaded or unloaded Ad-hMSCs on U87MG (*left panel*) or GSCs (*right panel*) is compared with the growth inhibition exerted by direct PTX treatment (scaled 1:2 dilutions) on U87MG (*left panel*) and GSCs (*right panel*). Mean ± SD are shown. (TIF 620 kb)
Additional file 2: Figure S2. In vitro coculture of U87MG and GSCs with PTX-loaded or unloaded Ad-hMSCs. Coculturing tumor cells with PTX-Ad-hMSCs, but not with Ad-hMSCs, results in a strong cytotoxic effect evidenced by an increase in the number of altered spindles (*white arrowhead*) and multinucleated tumor cells (*yellow arrows*) in both U87MG (*upper panel*) and GSCs (*lower panel*) (**a**). Mono/multipolar spindles significantly increase both in U87MG and in GSCs at 24 h of coculture with PTX-AdMSCs (****p* < 0.0001) (**b**). At 48 h of coculture, the PTX-Ad-hMSCs induce a significant increase in the percentage of multinucleated cells of both U87MG and GSCs (****p* < 0.0001) (**c**). *Scale bar* = 25 μm. (TIF 9888 kb)
Additional file 3: Figure S3. Time course of hMSC homing to brain U87MG xenografts and to lungs of matched rats. Fluorescence microphotographs of GFP-expressing U87MG brain xenografts (*left panel*) by 1 h and 24 h after injection of mCherry BM-hMSCs into the common carotid artery with ligation of the external carotid artery in athymic rats. The lungs of matched rats were also assessed by fluorescence microscopy at the same time points (*right panel*). *Scale bars* = 80 μm for left pictures of panels; *scale bars* = 25 μm for right pictures of panels. (*TIF 9508 kb*)

## Abbreviations

Ad-hMSC: Human adipose-derived mesenchyma stem/stromal cell
BBB: Blood-brain barrier
bFGF: Basic fibroblast growth factor
BM: Bone marrow
BM-hMSC: Human bone marrow-derived mesenchymal stem/stromal cell
CM: Conditioned medium
DAPI: 4′,6-Diamidino-2-phenylindole
DMEM: Dulbecco’s modified Eagle’s medium
FBS: Fetal bovine serum
GBM: Glioblastoma
GFP: Green fluorescent protein
GSC: Glioma stem cell
hMSC: Human mesenchymal stem/stromal cell
IMDM: Iscove’s modified Dulbecco’s medium
MSC: Mesenchymal stem/stromal cell
NDS: Normal donkey serum
PBS: Phosphate-buffered saline
PE: Phycoerythrin
PTX: Paclitaxel
VEGF: Vascular endothelial growth factor
