# Supplementary figures and images for: Human mesenchymal stromal cells inhibit tumor growth in orthotopic glioblastoma xenografts
Source: Stem Cell Res Ther. 2017 Mar 9;8:53. doi: 10.1186/s13287-017-0516-3 (PMC5345323; doi:10.1186/s13287-017-0516-3)

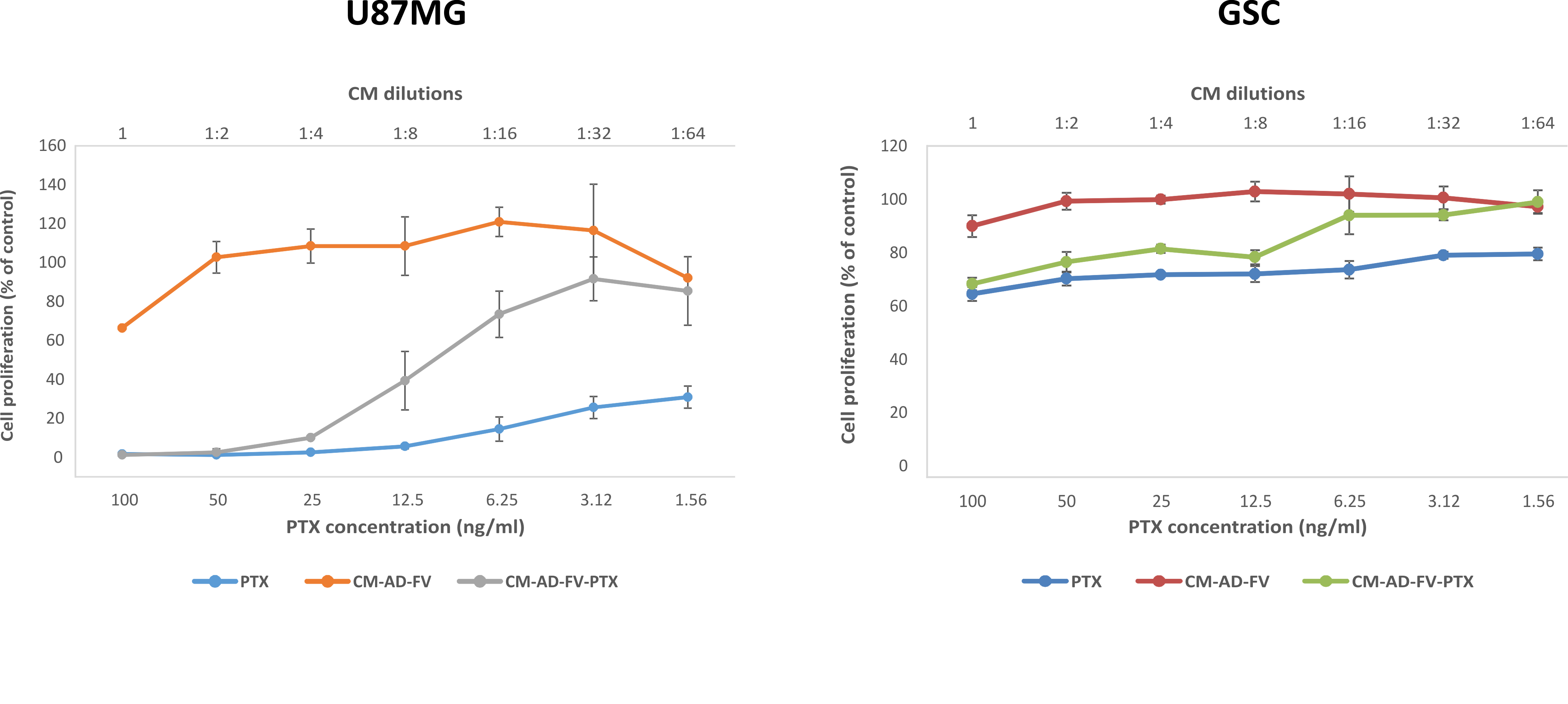

Supplement: Additional file 1: Figure S1. — In vitro U87MG and GSC cell viability after treatment with CM from PTX-loaded Ad-hMSCs. The kinetic of growth inhibition induced by serial 1:2 dilutions of CM from PTX-loaded or unloaded Ad-hMSCs on U87MG (left panel) or GSCs (right panel) is compared with the growth inhibition exerted by direct PTX treatment (scaled 1:2 dilutions) on U87MG (left panel) and GSCs (right panel). Mean ± SD are shown. (TIF 620 kb) [file 13287_2017_516_MOESM1_ESM.tif]

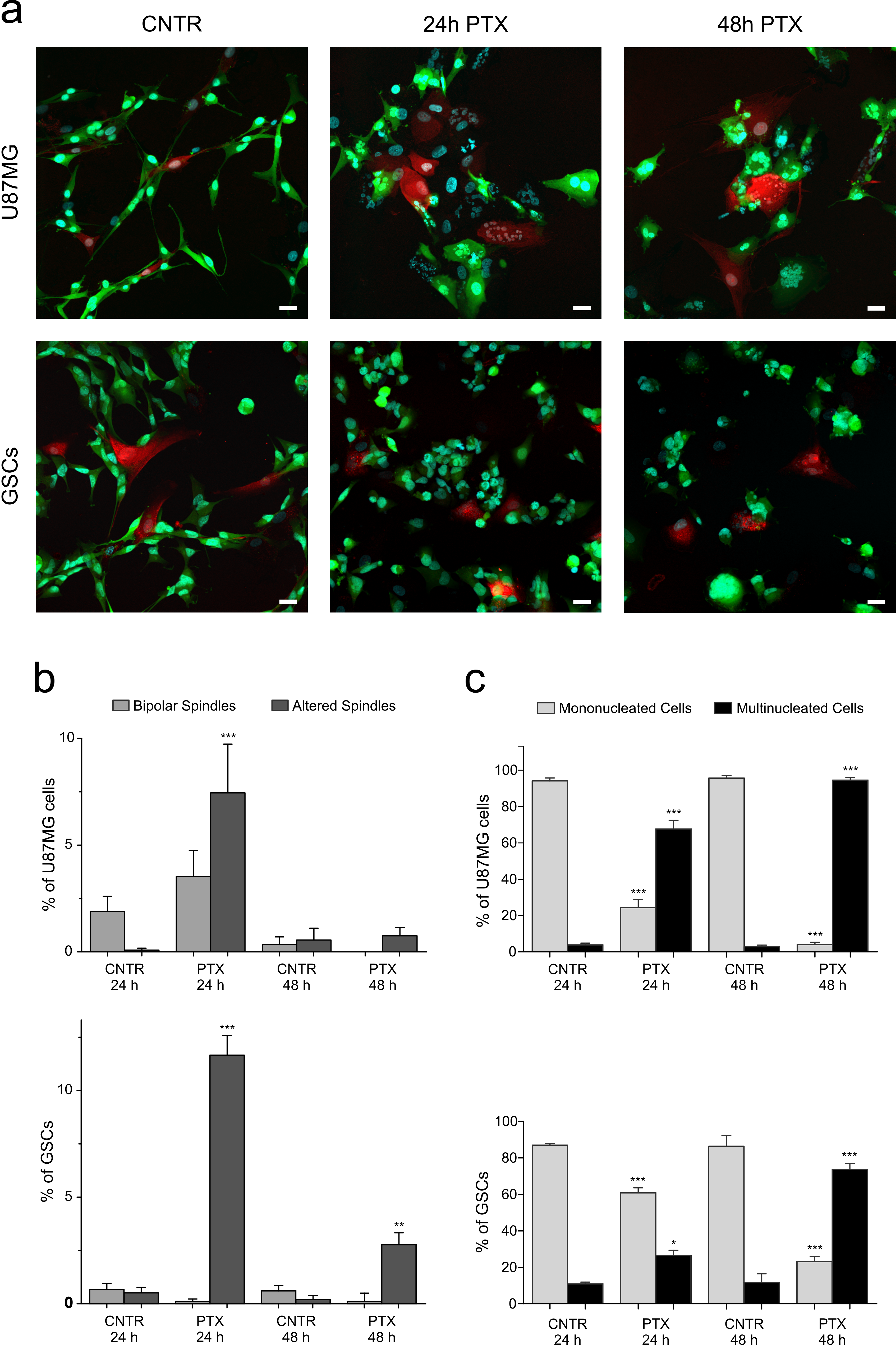

Supplement: Additional file 2: Figure S2. — In vitro coculture of U87MG and GSCs with PTX-loaded or unloaded Ad-hMSCs. Coculturing tumor cells with PTX-Ad-hMSCs, but not with Ad-hMSCs, results in a strong cytotoxic effect evidenced by an increase in the number of altered spindles (white arrowhead) and multinucleated tumor cells (yellow arrows) in both U87MG (upper panel) and GSCs (lower panel) (a). Mono/multipolar spindles significantly increase both in U87MG and in GSCs at 24 h of coculture with PTX-AdMSCs (***p < 0.0001) (b). At 48 h of coculture, the PTX-Ad-hMSCs induce a significant increase in the percentage of multinucleated cells of both U87MG and GSCs (***p < 0.0001) (c). Scale bar = 25 μm. (TIF 9888 kb) [file 13287_2017_516_MOESM2_ESM.tif]

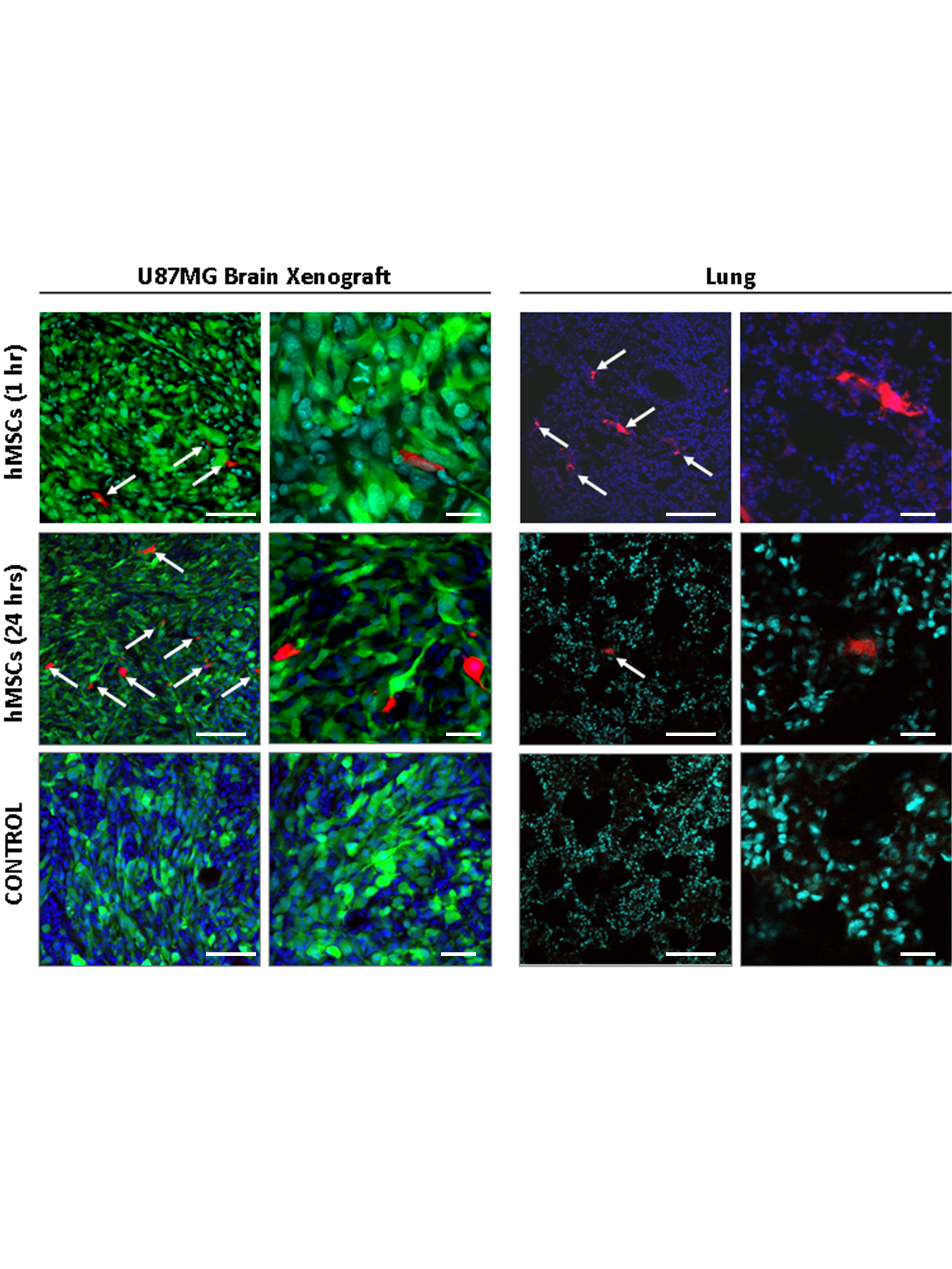

Supplement: Additional file 3: Figure S3. — Time course of hMSC homing to brain U87MG xenografts and to lungs of matched rats. Fluorescence microphotographs of GFP-expressing U87MG brain xenografts (left panel) by 1 h and 24 h after injection of mCherry BM-hMSCs into the common carotid artery with ligation of the external carotid artery in athymic rats. The lungs of matched rats were also assessed by fluorescence microscopy at the same time points (right panel). Scale bars = 80 μm for left pictures of panels; scale bars = 25 μm for right pictures of panels. (TIF 9508 kb) [file 13287_2017_516_MOESM3_ESM.tif]
